# Fishing on the Amazon

**Published:** 1882-03

**Authors:** 


### FISHING ON THE AMAZON.

Much attention has lately been given to the wonders of the great river Amazon, or "the Amazons," as the people there call it. Its whole valley abounds in streams that help to make up the entire volume of waters. These spread out into lakes, lagoons and swamps, that extend over large regions of country. This is especially so in the rainy seasons or flood times.

The channels and lakes are abundantly supplied with fishes. Even large fishes are often left in the swamp lakes and streams when the water is low. A hundred different kinds of fish can be bought in the markets of Rio, many of which come from the Amazon.

Those most valued are "piranhas" and "pirarucus." They are the largest, while there are numerous smaller varieties. The Indians catch the latter with hooks and lines or shoot them with arrows. But the larger fish are speared with a kind of trident. The men and even small boys acquire great skill in the use of these implements.

In the summer months the people come by hundreds to the lakes and channels to fish for the great "pirarucu," and to prepare the fish much as codfish is prepared by the northern fishermen. Some of these fish are seven or eight feet in length. They are first dressed and cut into wide, thin slices. These are well rubbed with salt and hung on poles to dry in the sun. The slices are taken under cover every night and carried out again in the morning. The stranger does not at once relish this dried fish, yet it is the standard flesh food of all the poorer classes throughout a large part of Brazil. During the fishing season the people build and live in little huts along the shores. Traders, in canoes, come with a stock of cheap wares to barter for the fish. Thus a trading community is formed, which breaks up with the January floods. The "piranhas" are much prized and are easily caught, from a bit of salt meat to a bather's toe. Boys thrash the water with poles to attract these fishes.

The Tupi word "piranha" is a contraction of "pira sainha," meaning "toothed fish." The same word is used by the Indians to describe a pair of scissors. There are several species of these savage "piranhas," some being more than two feet long. They think nothing of biting an ounce or so of flesh from a man's leg. People are sometimes killed by them. Hence Brazilians are shy of going into these lakes and streams if they suspect the presence of these fish. The fishermen claim that "piranhas" will gather in schools against the larger fish and attack them. If one of their number is at all wounded by mistake he is mercilessly set upon and devoured by his companions.

It is useless to try to use nets where this fish is found. They would spoil a net in a few minutes.

---

One in every six of our 50,132,866 population is a church member.

The next use of the Mayflower, after her memorable voyage to America, was to carry a cargo of slaves to the West Indies.

To think we are able is almost to be so; to determine upon attainments is frequently attainment itself. Thus earnest resolution has often seemed to have about it almost a savor of omnipotence.

Drink for Sick Stomach.—The following drink for relieving sickness of the stomach was introduced by Dr. Halahan, and it is said to be very palatable and agreeable: Beat up one egg very well, say for twenty minutes, then add fresh milk, one pint; water, one pint; sugar, to make it palatable; boil and let it cool; drink when cold. If it become curd and whey it is useless.

A charming old pedant in the country on learning that a favorite pupil of his had been taken upon the staff of a Boston paper, wrote to the editor-in-chief concerning the young man: "If he should have a career I shall be very happy in thinking that the spark which I have watered contained in it the germ of a structure destined to soar and elevate with its radiance your privileged readers." He also advised the editor to "give the young man a hint that may quench the seeds of ambition ere yet they swell to a gale that will take the bits between its teeth and dazzle by its clamor."—*Boston Courier*.

